# Alteration of PD-L1 (SP142) status after neoadjuvant chemotherapy and its clinical significance in triple-negative breast cancer

**DOI:** 10.1007/s10549-024-07359-x

**Published:** 2024-05-16

**Authors:** Ji Won Woo, Eun Kyung Han, Koung Jin Suh, Se Hyun Kim, Jee Hyun Kim, So Yeon Park

**Affiliations:** 1 Department of Pathology, Seoul National University Bundang Hospital, Seoul National University College of Medicine, 82, Gumi-ro 173 Beon-gil, Bundang-gu, Seongnam, Gyeonggi 13620 Republic of Korea; 2 Division of Hematology and Medical Oncology, Department of Internal Medicine, Seoul National University Bundang Hospital, Seoul National University College of Medicine, Seongnam, Gyeonggi Republic of Korea

**Keywords:** Triple-negative breast cancer, PD-L1, SP142, Neoadjuvant chemotherapy

## Abstract

**Purpose:**

The tumor immune microenvironment can change after neoadjuvant chemotherapy (NAC) for triple-negative breast cancer (TNBC). We aimed to investigate the effects of NAC on PD-L1 (SP142) status and its clinical significance in TNBC.

**Methods:**

Paired samples of biopsy and resection specimens were collected from 182 patients with TNBC before and after NAC. PD-L1 (SP142) expression in immune cells in pre- and post-NAC breast cancer samples and the changes between them were analyzed, along with their relationships with the clinicopathological features and clinical outcomes of the patients.

**Results:**

Of the 182 patients, 61 (33.5%) achieved pathologic complete response (pCR) after NAC. PD-L1 (SP142) positivity, defined as immune cell staining in ≥ 1% of tumor area, was a predictor for pCR. PD-L1-positive immune cells significantly increased after NAC (2.8% to 5.2% on average) in 109 patients with measurable residual disease. Alteration of PD-L1 status was observed in 24 (22.0%) of the 109 patients with measurable residual tumors after NAC, and all PD-L1 status-converted patients, except one, revealed negative-to-positive conversion. Regarding chemotherapeutic agents, the use of platinum agents was associated with a significant increase in PD-L1-positive immune cells after NAC. In survival analyses, a positive PD-L1 status after NAC and increase of PD-L1-positive immune cells after NAC were associated with better recurrence-free survival of the patients.

**Conclusion:**

PD-L1 (SP142) status changes after NAC, mostly as a positive conversion. As PD-L1 (SP142) status can convey prognostic and predictive information, it needs to be tested before and after NAC.

**Supplementary Information:**

The online version contains supplementary material available at 10.1007/s10549-024-07359-x.

## Introduction

Triple-negative breast cancer (TNBC) accounts for 10–20% of all breast cancers [1]. Despite its aggressive behavior and poor clinical outcome [1], the lack of targeted therapies, such as endocrine and human epidermal growth factor receptor 2 (HER2)-targeted therapies, makes it more complicated to control this disease. Although it has been a challenge to determine novel therapeutic breakthroughs, immunotherapy, which boosts the immune system to fight cancer, has recently received increasing attention [2].

One of the well-known targets of immunotherapy is the programmed death 1 (PD-1)/programmed death-ligand 1 (PD-L1) axis, and two drugs targeting this axis have recently been approved for TNBC. Atezolizumab, a PD-L1 inhibitor, gained accelerated approval from the US Food and Drug Administration for the treatment of locally advanced or metastatic TNBC [3]; however, it was withdrawn after a subsequent trial failed to prove its superiority in survival [4]. Pembrolizumab, which targets PD-1, has been approved for the treatment of recurrent or metastatic [5] and high-risk early-stage TNBC [6]. Each drug uses a different antibody as a companion diagnostics: PD-L1 (SP142) [7] and PD-L1 (22C3) [8]. The interpretation methods of these antibodies also differ.

Neoadjuvant chemotherapy (NAC) is the standard treatment for locally advanced breast cancer [9]. NAC can affect the tumor immune microenvironment in several ways [10]. Axelrod et al. [11] demonstrated that changes in tumor immunity after NAC were more common in TNBCs compared with non-TNBCs. The tumor immune microenvironment is significantly altered after chemotherapy, creating opportunities for subsequent immunotherapy [12]. Moreover, these alterations differ according to specific tumor subtypes, types of antibody used, and evaluation methods applied and may be affected by the specific regimens of chemotherapy [12]. Several studies have evaluated alterations in PD-L1 status after NAC in breast cancer [13–17]; however, the results are inconsistent and limited by the inclusion of various breast cancer subtypes and small sample sizes. Furthermore, heterogeneous PD-L1 antibodies have been used in these studies.

Tumor-infiltrating lymphocytes (TILs) are well-established predictive [18, 19] and prognostic factors [16, 20, 21] for TNBCs. TILs reflect the host immune response against tumors and can be easily assessed using hematoxylin and eosin-stained sections. The combination of PD-L1 and TILs can convey prognostic information [13–17].

In this study, we aimed to investigate the effects of NAC on the PD-L1 (SP142) status of tumors using a large number of paired TNBC samples before and after NAC in relation to TIL levels and chemotherapeutic regimens. We also evaluated the alteration in PD-L1 (SP142) status after NAC and its association with the clinicopathological features of tumors and the clinical outcomes of patients. In addition, PD-L1 (SP142) and PD-L1 (22C3) was compared and the change of PD-L1 (22C3) status after NAC was evaluated in a small subset of patients.

## Methods

### Patient selection

Patients with advanced or high-risk early TNBC who received NAC followed by surgery and underwent PD-L1 (SP142) immunohistochemical staining at Seoul National University Bundang Hospital between 2015 and 2022 were enrolled. TNBC was defined as estrogen receptor (ER)-negative, progesterone receptor (PR)-negative [22] and HER2-negative [23] breast cancer in a pre-NAC biopsy sample. In total, 182 patients with TNBC were included in this study. Of the 182 patients, 35 had PD-L1 (22C3) immunohistochemical data as well. A total of 109 patients with measurable residual disease and PD-L1 (SP142) status data after NAC were used for paired comparison.

### Clinicopathological information

Clinical information of the patients was obtained through review of medical records, and the following information was recorded: age at diagnosis, sex, initial clinical T and N stages, chemotherapeutic regimen, cycle of NAC, tumor recurrence, site of recurrence, patient survival, and follow-up period. Pathologic information was obtained from the pathology reports and slide reviews. Pathologic response to NAC was evaluated using the Residual Cancer Burden (RCB) class [24]. Pathologic complete response (pCR) was defined as the complete disappearance of all invasive tumor cells from the breast and regional lymph nodes, regardless of the presence of residual ductal carcinoma in situ in the breast [25].

### Evaluation of PD-L1 (SP142) and PD-L1 (22C3)

The PD-L1 (SP142) assay (Ventana Medical Systems, Tucson, AZ, USA) was performed on 4-μm-thick sections from formalin-fixed paraffin-embedded tissue blocks using the OptiView DAB IHC detection kit (Ventana Medical Systems) and OptiView Amplification Kit (Ventana Medical Systems) on a BenchMark ULTRA platform (Ventana Medical Systems), according to the manufacturer’s instructions. The presence of discernible PD-L1 staining of any intensity in tumor-infiltrating immune cells was scored as a percentage, with the tumor area as the denominator. The tumor area was defined as the space occupied by the tumor cells and the associated intratumoral and contiguous peritumoral stroma. Immune cell staining in ≥ 1% of tumor area was classified as positive [7].

PD-L1 IHC 22C3 pharmDx assay (Agilent, Santa Clara, CA, USA) was performed using the EnVision FLEX visualization system (Agilent) on an Autostainer Link 48 system (Agilent), as per the manufacturer’s instructions. Combined positive score (CPS) was calculated by dividing the number of PD-L1 (22C3)-stained cells (both tumor cells and immune cells) by the total number of viable tumor cells and multiplying the value by 100, and CPS of 10 or more was classified as positive [8].

### Evaluation of TILs

TILs were evaluated by two pathologists (JWW and EKH) following the guidelines provided by International Immune-Oncology Biomarker Working Group [26, 27]. For the analysis, TIL status was categorized into three groups: low TIL (TIL < 10%), moderate TIL (10% ≤ TIL < 50%), and high TIL (TIL ≥ 50%). In the resected samples after NAC, TIL evaluation was performed in 109 patients, excluding cases not suitable for evaluation: no residual tumor, residual in situ, or microinvasive carcinoma only.

### Statistical analyses

The Statistical Package for the Social Sciences version 25.0 (IBM Corporation, Armonk, NY, USA) was used for the statistical analyses. Paired sample t-test was used to compare the proportion of PD-L1-positive immune cells before and after NAC. The chi-square or Fisher’s exact test was used to evaluate the relationship between categorical variables. Spearman’s rank correlation test was used to assess the correlation between two variables. Survival curves were drawn using the Kaplan–Meier method, and *p-*values were calculated using the log-rank test. The Cox proportional hazards model was used for multivariate analysis using a backward stepwise selection method with covariates associated with patient survival in univariate analyses. All reported *p-*values were two-sided, and *p-*value < 0.05 was considered statistically significant.

## Results

### Clinicopathological characteristics of patients

Of the 182 patients, 61 (33.5%) had clinical stage II disease, and 116 (63.7%) had stage III disease. Five (2.7%) patients had clinical stage I disease. Most of the patients (*n* = 144, 79.1%) received anthracycline and taxane-based chemotherapy. Six patients (3.3%) received neoadjuvant pembrolizumab in combination with chemotherpay. Patients underwent breast surgery 3–4 weeks after their last chemotherapy cycle. The clinicopathological characteristics of the patients before NAC are summarized in Supplementary Table S1.

### PD-L1 (SP142) as a predictor of pathologic complete response

After completion of NAC, 61 (33.5%) of the 182 patients achieved pCR. Nineteen (10.4%) patients had RCB I, 74 (40.7%) had RCB II, and 28 (15.4%) had RCB III after NAC. Table 1 shows the relationship between pre-NAC variables and chemotherapy response indices. Among the variables analyzed, PD-L1 (SP142)-positive status predicted pCR along with a low clinical T stage and a high Ki-67 index. PD-L1-positive status was significantly more frequent in tumors showing pCR compared with those not achieving pCR (59.0% vs. 37.2%, *p* = 0.005). PD-L1 positivity also correlated with low RCB classes (RCB 0 and RCB I) (*p* = 0.002). Patients with a low clinical T stage and a high Ki-67 index also showed low RCB classes.

**Table 1:** Relationship between clinicopathological characteristics of tumor and chemo-response indices

Clinicopathological characteristics before NAC	Pathologic complete response		*p *value	RCB class		*p *value
	Achieved (*n* = 61)	Not achieved (*n* = 121)		RCB 0 & I (*n* = 80)	RCB II & III (*n* = 102)	
Age			0.105			0.140
< 50 years	38 (62.3)	60 (49.6)		48 (60.0)	50 (49.0)	
≥ 50 years	23 (37.7)	61 (50.4)		32 (40.0)	52 (51.0)	
cT category			0.035			0.002
T1 & T2	54 (88.5)	91 (75.2)		72 (90.0)	73 (71.6)	
T3 & T4	7 (11.5)	30 (24.8)		8 (10.0)	29 (28.4)	
cN category			0.117			0.154
N0	17 (27.9)	48 (39.7)		24 (30.0)	41 (40.2)	
N1-N3	44 (72.1)	73 (60.3)		56 (70.0)	61 (59.8)	
Clinical stage			0.368			0.949
I & II	21 (34.4)	50 (41.3)		31 (38.8)	40 (39.2)	
III	40 (65.6)	71 (58.7)		49 (61.3)	62 (60.8)	
Histologic grade			0.067			0.084
II	8 (13.1)	30 (24.8)		12 (15.0)	26 (25.5)	
III	53 (86.9)	91 (75.2)		68 (85.0)	76 (74.5)	
Ki-67 index			0.003			< 0.001
< 50%	13 (21.3)	53 (43.8)		16 (20.0)	50 (49.0)	
≥ 50%	48 (78.7)	68 (56.2)		64 (80.0)	52 (51.0)	
TIL category			0.100			0.081
< 10%	24 (39.3)	68 (56.2)		33 (41.3)	59 (57.8)	
≥ 10% and < 50%	24 (39.3)	34 (28.1)		31 (38.8)	27 (26.5)	
≥ 50%	13 (21.3)	19 (15.7)		16 (20.0)	16 (15.7)	
PD-L1 (SP142) status			0.005			0.002
Negative	25 (41.0)	76 (62.8)		34 (42.5)	67 (65.7)	
Positive	36 (59.0)	45 (37.2)		46 (57.5)	35 (34.3)	

Number in parenthesis indicates percentage

*NAC* neoadjuvant chemotherapy, *TIL* tumor-infiltrating lymphocytes, *RCB* residual cancer burden

*p*-value was calculated by Chi-square or Fisher’s exact test

### Alteration of PD-L1 (SP142) after NAC

PD-L1 (SP142) status was evaluated in resection samples after NAC in 109 patients with measurable residual disease, and these data were used for paired comparison. The proportion of PD-L1-positive immune cells increased from an average of 2.8% to 5.2% after NAC (*p* = 0.020, Table 2). In respect to histologic subtype, invasive carcinoma of no special type showed an increase in the number of PD-L1-positive immune cells after NAC (3.1% to 5.9% on average; *p* = 0.022); however, metaplastic carcinoma did not show an increase in the number of PD-L1-positive immune cells after NAC (1.5% to 0.6% on average; *p* = 0.531). Increase in PD-L1-positive immune cells occurred in all TIL categories, and patients with low and moderate TIL before NAC showed a significant increase in PD-L1-positive immune cells (*p* = 0.034 and *p* = 0.013, respectively; Table 2).

**Table 2:** Alteration of PD-L1 (SP142)-positive immune cells after neoadjuvant chemotherapy according to tumor infiltrating lymphocyte category

TIL category before NAC	Proportion of PD-L1-positive immune cells (%)		*p* value
	Before NAC	After NAC	
Low-TIL (TIL < 10%)	0.5 (1.6)	2.1 (6.0)	0.034
Moderate-TIL (10% ≤ TIL < 50%)	2.0 (3.2)	6.1 (7.4)	0.013
High-TIL (TIL ≥ 50%)	12.1 (15.9)	14.9 (14.4)	0.598
Total	2.8 (7.8)	5.2 (9.4)	0.020

Number in parenthesis indicates standard deviation

*NAC* neoadjuvant chemotherapy, *TIL* tumor-infiltrating lymphocytes

*p*-value was calculated by paired sample *t*-test

**Fig. 1:**
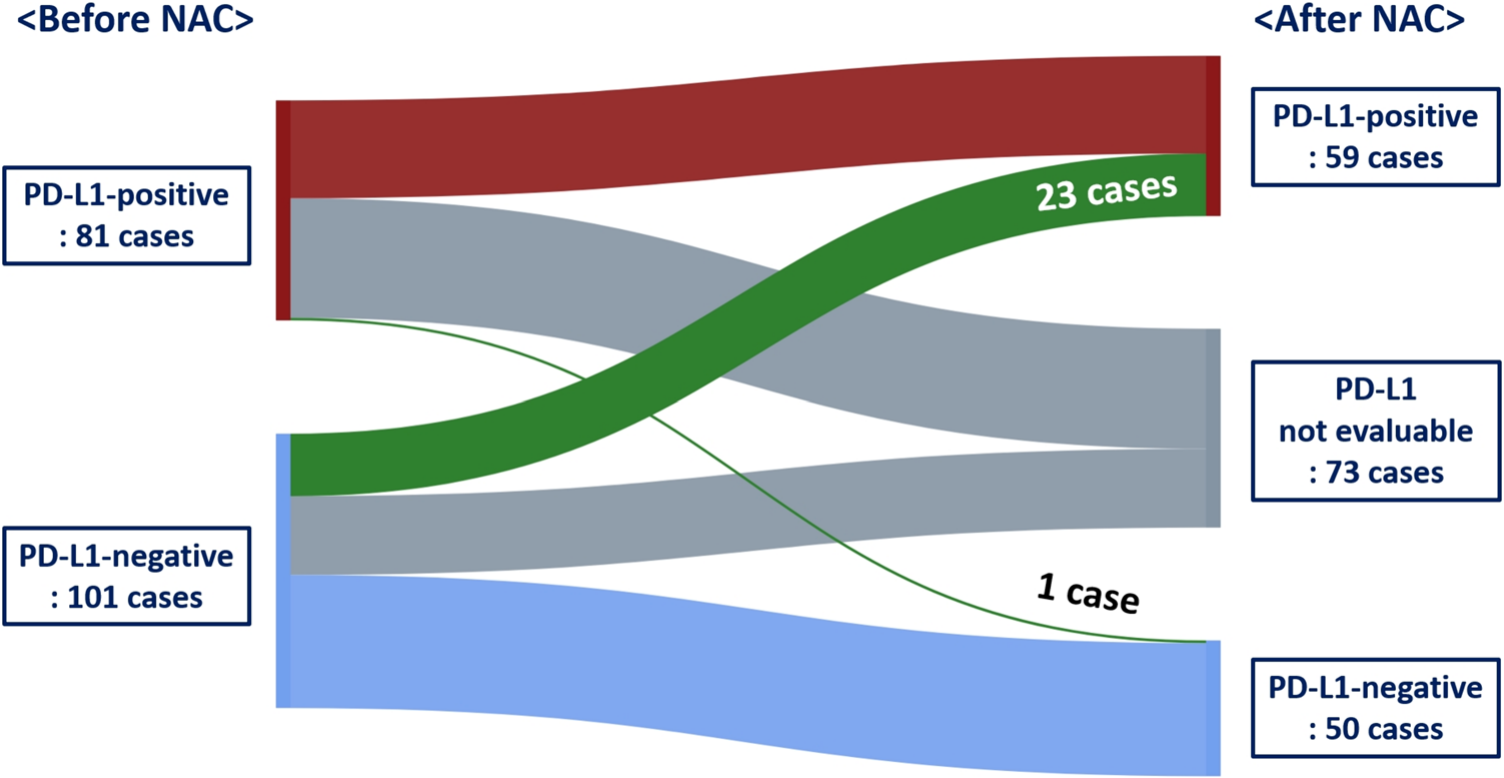
Sankey plot demonstrating PD-L1 (SP142) status conversion after neoadjuvant chemotherapy. Green color indicates PD-L1 conversion. In total, 23 cases of positive conversion and 1 case of negative conversion occurred. Red color means PD-L1 remained positive, blue color means PD-L1 remained negative, and gray color means cases that PD-L1 was not evaluable due to pathologic complete response or residual small cancer

**Fig. 2:**
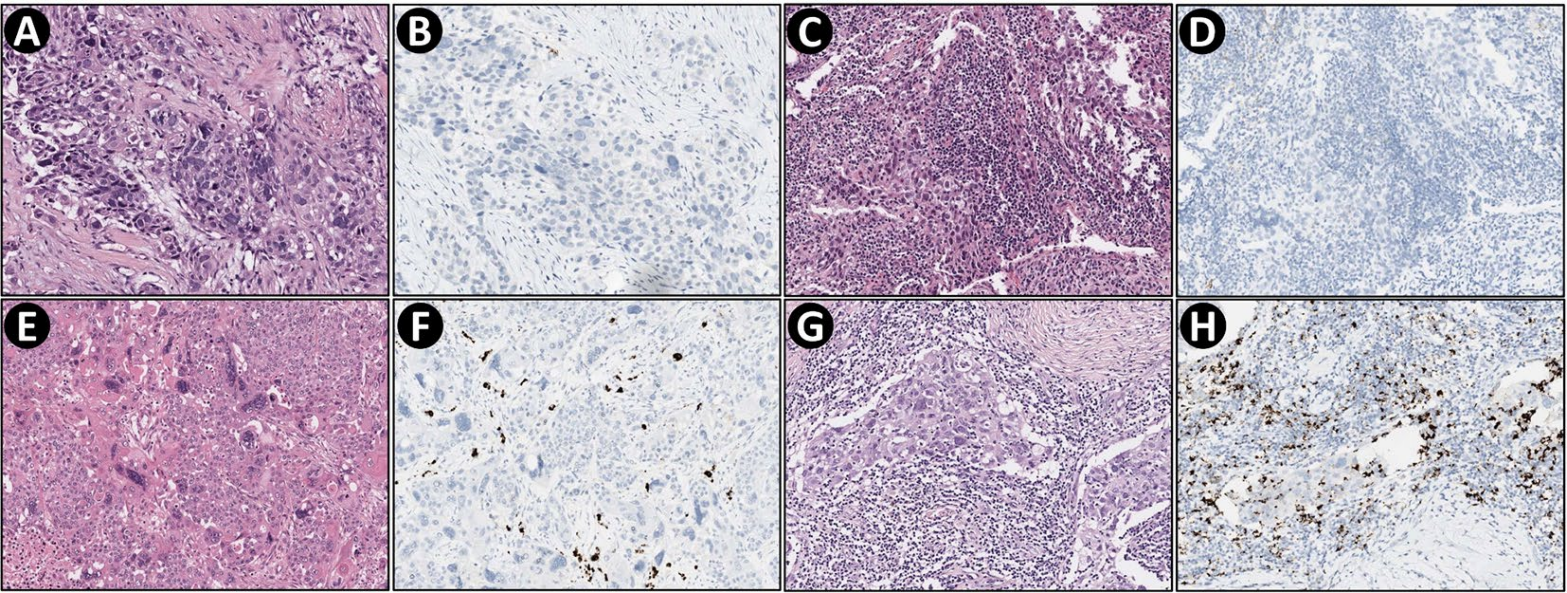
Representative images of PD-L1 (SP142)-positive conversion after neoadjuvant chemotherapy (NAC). Pre-NAC images from biopsy specimen are in the upper row, and post-NAC images from resection specimen are in the lower row. **A, B, E, F** Although tumor-infiltrating lymphocytes (TILs) did not increase, PD-L1-positive immune cells clearly increased and PD-L1 status changed from negative to positive. **C, D, G, H** High TIL is observed before and after NAC. Although PD-L1-positive immune cells are hardly detected before NAC (< 1% of tumor area), they are abundant after NAC

Collectively, changes in PD-L1 status (negative or positive conversion) after NAC were observed in 24 of the 109 patients (22.0%) (Fig. 1). Of the 24 patients, 23 (95.8%) showed positive conversion (Fig. 2). The case showing negative conversion revealed a decreased number of PD-L1-positive immune cells from 10.0% to < 1%, and it was metaplastic carcinoma with a poor response to NAC (RCB III).

Although the PD-L1 status significantly changed after NAC, the TIL category did not significantly change, showing a moderate positive correlation between the TIL categories before and after NAC (rho = 0.429, *p* < 0.001; Supplementary Table S2). However, both decreased [31 of 109 (28.4%) patients] and increased TIL [11 of 109 (10.1%) patients] were observed after NAC in some patients. The PD-L1 and TIL category distributions before and after NAC are summarized in Supplementary Table S3. They also showed moderate positive correlations (before NAC, rho = 0.467, *p* < 0.001; after NAC, rho = 0.473, *p* < 0.001).

### Alteration of PD-L1 (SP142) in patients treated with platinum-based agents

**Fig. 3:**
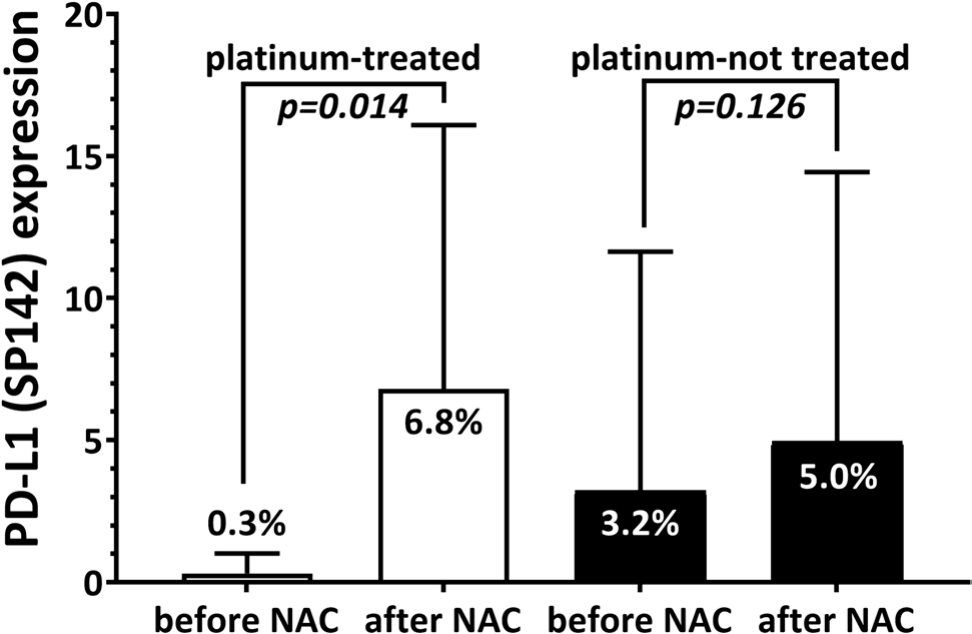
Change of PD-L1 (SP142) status after neoadjuvant chemotherapy (NAC) showed difference according to chemotherapeutic regimens. Among the 26 patients treated with platinum-based agents, 16 had residual disease suitable for post-NAC PD-L1 evaluation. After platinum-based NAC, PD-L1-positive immune cells increased from 0.3% to 6.8% in average (*p* = 0.014). Patients treated without platinum-based agents showed increased PD-L1-positive immune cells with a borderline statistical significance (from average 3.2% to 5.0%, *p* = 0.126). *p*-values were calculated using the paired sample *t*-test

Most of the patients were treated with anthracycline and taxane-based regimens; however, 26 (14.3%) of the 182 patients received additional platinum agents for NAC. These patients showed a significant difference in the changes in PD-L1 (SP142)-positive immune cells after NAC. PD-L1-positive immune cells dramatically increased after platinum-based chemotherapy, from 0.3% to 6.8% (*p* = 0.014), a more than 20-fold increase, whereas the other group showed less than a twofold increase (from 3.2% to 5.0%, *p* = 0.126) (Fig. 3).

Increase in PD-L1-positive immune cells and positive conversion of PD-L1 status after NAC were also common in platinum agent-treated patients. However, their clinical significance was not determined, probably owing to the small number of patients (Supplementary Table S4).

### Comparison of PD-L1 (SP142) and PD-L1 (22C3)

Next, comparative analysis was performed using 35 cases with both PD-L1 (SP142) and PD-L1 (22C3) data. The proportion of PD-L1 (SP142)-positive immune cells and the CPS of PD-L1 (22C3) was highly correlated in both pre-NAC biopsy (rho = 0.702, *p* < 0.001) and post-NAC resection specimen (rho = 0.821, *p* < 0.001). The status of PD-L1 (SP142) and PD-L1 (22C3) also showed moderate to strong correlations (before NAC, rho = 0.663, *p* < 0.001; after NAC, rho = 0.716, *p* < 0.001).

PD-L1 (22C3) positive rate was significantly higher in pCR group compared to non-pCR group (81.8% vs. 41.7%, *p* = 0.035). In the 21 cases where paired comparison was possible, the CPS of PD-L1 (22C3) increased from an average of 12.5 to 15.9 after NAC, but without statistical significance (*p* = 0.257). The change in PD-L1 (22C3) status after NAC were found in 3 of the 21 patients (14.3%), and all of them showed positive conversion.

### PD-L1 (SP142) as a prognostic factor

**Fig. 4:**
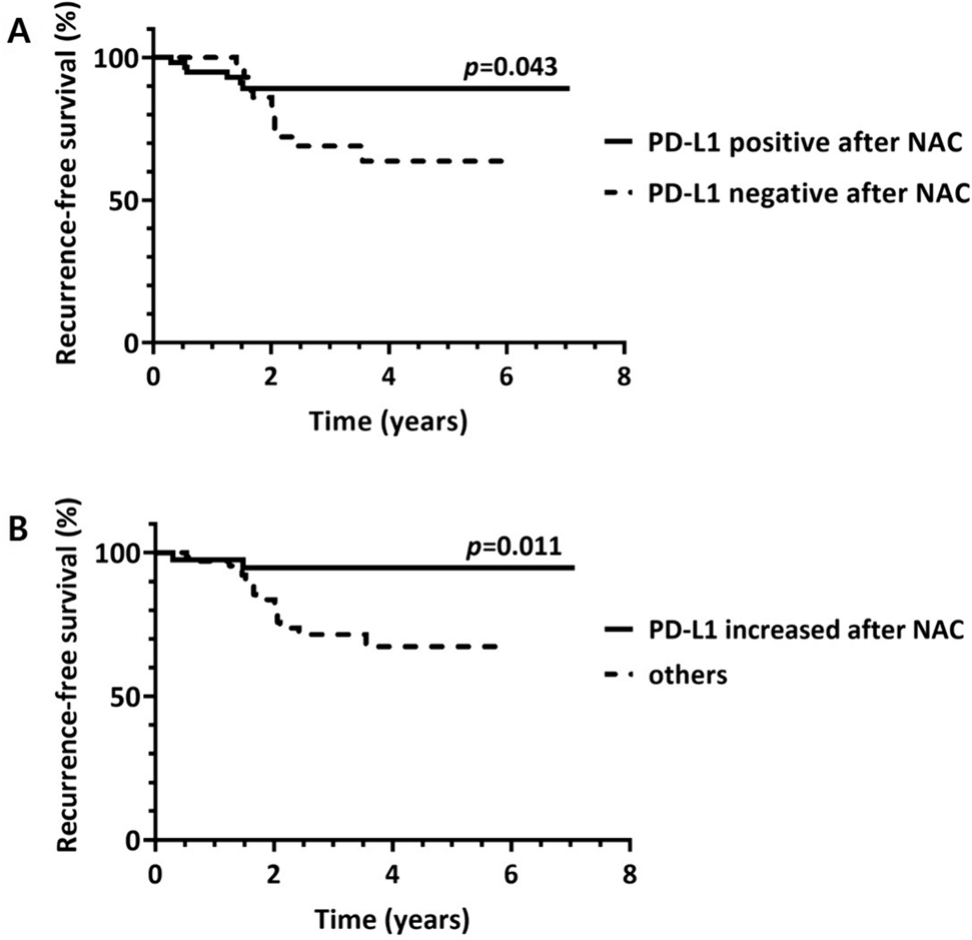
**A** Patients with positive PD-L1 (SP142) status after neoadjuvant chemotherapy (NAC) demonstrated better recurrence-free survival (*p* = 0.043, log-rank test). **B** Patient group with increased PD-L1 (SP142)-positive immune cells after NAC showed better recurrence-free survival than the other groups (*p* = 0.011, log-rank test)

The mean follow-up period of the 182 patients was 2.85 years with 22 (12.1%) cases of recurrence and 7 (3.8%) cases of death due to disease progression. Although positive PD-L1 (SP142) status before NAC was associated with better recurrence-free survival (RFS) of the patients with borderline significance (*p* = 0.064), positive PD-L1 status after NAC was significantly correlated with increased RFS of the patients (*p* = 0.043; Fig. 4). PD-L1 statuses before and after NAC were not significantly associated with overall survival (OS) of the patients (*p* = 0.319 and *p* = 0.326, respectively). TIL statuses before and after NAC were not significantly correlated with RFS (*p* = 0.327 and *p* = 0.053, respectively) or OS (*p* = 0.209 and *p* = 0.530, respectively).

Among the 109 patients with residual disease available for PD-L1 evaluation after NAC, 41 showed an increase in PD-L1-positive immune cells after NAC. These patients demonstrated better RFS (*p* = 0.011; Fig. 4) and showed a correlation with a low clinical T stage before treatment, negative node status after NAC, frequent TIL infiltration before and after NAC, and low RCB class (Table 3).

**Table 3:** Clinicopathological characteristics of tumors according to change of PD-L1 (SP142)-positive immune cells after neoadjuvant chemotherapy

Clinicopathological characteristics	Change of PD-L1-positive immune cells after NAC		*p* value
	Not increased (*n* = 68)	Increased (*n* = 41)	
Pre-NAC variables			
Age			0.902
< 50 years	34 (50.0)	21 (51.2)	
≥ 50 years	34 (50.0)	20 (48.8)	
cT category			0.028
T1 & T2	45 (66.2)	35 (85.4)	
T3 & T4	23 (33.8)	6 (14.6)	
cN category			0.707
N0	29 (42.6)	19 (46.3)	
N1- 3	39 (57.4)	22 (53.7)	
Clinical stage			0.217
I & II	25 (36.8)	20 (48.8)	
III	43 (63.2)	21 (51.2)	
Histologic grade			0.252
II	20 (29.4)	8 (19.5)	
III	48 (70.6)	33 (80.5)	
Ki-67 index			0.746
< 50%	31 (45.6)	20 (48.8)	
≥ 50%	37 (54.4)	21 (51.2)	
TIL category			0.001
< 10%	49 (72.1)	14 (34.1)	
≥ 10% and < 50%	12 (17.6)	16 (39.0)	
≥ 50	7 (10.3)	11 (26.8)	
PD-L1 status			0.088
Negative	49 (72.1)	23 (56.1)	
Positive	19 (27.9)	18 (43.9)	
Post-NAC variables			
ypT category			0.155
T1 & T2	63 (92.6)	41 (100.0)	
T3 & T4	5 (7.4)	0 (0.0)	
ypN category			0.004
N0	36 (52.9)	33 (80.5)	
N1-N3	32 (47.1)	8 (19.5)	
Pathologic stage after NAC			0.051
I & II	51 (75.0)	37 (90.2)	
III	17 (25.0)	4 (9.8)	
Lymphovascular invasion			0.109
Absent	45 (66.2)	33 (80.5)	
Present	23 (33.8)	8 (19.5)	
Ki-67 index			0.137
< 10%	26 (38.2)	109 (24.4)	
≥ 10%	42 (61.8)	31 (75.6)	
TIL category			< 0.001
< 10%	58 (85.3)	19 (46.3)	
≥ 10% and < 50%	10 (14.7)	18 (43.9)	
≥ 50%	0 (0.0)	4 (9.8)	
PD-L1 status			< 0.001
Negative	50 (73.5)	0 (0.0)	
Positive	18 (26.5)	41 (100.0)	
RCB class			0.039
RCB I	4 (5.9)	7 (17.1)	
RCB II	42 (61.8)	28 (68.3)	
RCB III	22 (32.4)	6 (14.6)	

Number in parenthesis indicates percentage

*NAC* neoadjuvant chemotherapy, *TIL* tumor-infiltrating lymphocytes, *RCB* residual cancer burden

*p*-value was calculated by Chi-square or Fisher’s exact test

In the multivariate analysis using pre-NAC variables and pCR, young age (< 50 years), high clinical stage, and non-pCR were identified as independent poor prognostic factors (*p* = 0.005, *p* = 0.026, and *p* = 0.017, respectively; Table 4). When using post-NAC variables, only high pathologic stage after NAC remained an independent poor prognostic factor (*p* < 0.001, Table 4*).*

**Table 4:** Univariate and multivariate analyses of recurrence-free survival

Variable	Category	Univariate analysis			Multivariate analysis		
		HR	95% CI	*p *value	HR	95% CI	*p *value
Pre-NAC variables and pCR							
Age	< 50 years vs. ≥ 50 years	3.2	1.2–8.6	0.024	4.3	1.6–11.7	0.005
Clinical stage	III vs. I & II	2.9	1.0–8.5	0.055	3.4	1.2–10.2	0.026
Histologic grade	III vs. II	1.1	0.4–3.4	0.806	–	–	–
Ki-67 index	< 50% vs. ≥ 50%	1.0	0.4–2.5	0.944	–	–	–
TIL category	< 10% vs. ≥ 10%	1.8	0.8–4.3	0.176	1.6	0.6–4.1	0.344
PD-L1 (SP142) status	Negative vs. positive	2.4	0.9–6.1	0.072	2.4	0.9–6.1	0.074
pCR	Not achieved vs. achieved	10.5	1.4–77.8	0.012	11.8	1.6–88.2	0.017
Post-NAC variables in cases with residual disease							
Pathologic stage	III vs. I & II	8.6	3.6–20.6	< 0.001	8.7	3.3–23.0	< 0.001
Histologic grade	III vs. II	1.1	0.4–2.9	0.798	–	–	–
Ki-67 index	≥ 10% vs. < 10%	1.7	0.6–5.02	0.328	–	–	–
LVI	Present vs. absent	6.3	2.5–16.1	< 0.001	2.3	0.8–6.9	0.126
TIL category	< 10% vs. ≥ 10%	3.8	0.9–16.6	0.073	1.5	0.3–7.5	0.643
RCB class	III vs. I & II	6.4	2.8–14.8	< 0.001	0.7	0.2–2.9	0.662
PD-L1 (SP142) status	Negative vs. positive	2.6	1.0–6.9	0.051	1.0	0.3–3.1	0.952
PD-L1 (SP142) change	Not increased vs. increased	5.4	1.3–23.5	0.024	3.1	0.7–13.8	0.145

*NAC* neoadjuvant chemotherapy, *TIL* tumor-infiltrating lymphocytes, *LVI* lymphovascular invasion, *HR* hazard ratio, *CI* confidence interval

*p*-value was calculated by Cox proportional hazards model

## Discussion

In this study, we evaluated the alteration of PD-L1 (SP142) status after NAC and its correlation with the clinicopathological features of tumors and clinical outcomes of patients. Several previous studies have compared the PD-L1 status before and after NAC in breast cancer [13–17]. However, only a few studies performed TNBC-specific analysis. Tomioka et al. [17] reported that PD-L1 (SP142)-positive tumor cells, but not PD-L1 (SP142) positive-immune cells showed a decreasing trend after NAC using 22 pairs of TNBC samples. Lee et al. [15] used PD-L1 (SP263) antibody to compare 34 pairs of TNBC samples before and after NAC. The median value of stromal PD-L1 increased by more than double, and the median value of PD-L1 expression in tumor cells slightly increased after NAC.

In the current study, 182 pairs of TNBC tissue samples before and after NAC were analyzed for PD-L1 (SP142) expression. After NAC, the number of PD-L1 (SP142)-positive immune cells increased from 2.8% to 5.2% on average, with a statistically significant difference. In particular, the number of PD-L1 (SP142)-positive immune cells dramatically increased after platinum agent treatment, as discussed in detail later. The conflicting results with previous studies probably come from differences in the PD-L1 antibody and scoring method used for analysis, large differences in the number of cases included in the study, and the inclusion of various subtypes of breast cancer. Although comparison with previous studies is not feasible for these reasons and a confirmatory study may be required, our study clearly demonstrated that PD-L1 (SP142)-positive immune cells increased after NAC. However, interestingly, metaplastic carcinoma showed no significant difference in the number of PD-L1 (SP142)-positive immune cells between pre- and post-NAC, and one of them showed negative conversion after NAC. Although this finding is limited by the small number of cases, it may reflect heterogeneity of the immune microenvironment according to subtypes of TNBCs.

CPS of PD-L1 (22C3) also showed an increase after NAC, though it was not statistically significant probably due to small sample size. As the proportion of PD-L1 (SP142)-positive immune cells and the CPS of PD-L1 (22C3) is highly correlated, it is supposed that PD-L1 (22C3) expression would increase after NAC in TNBCs. However, further confirmative studies about alteration of PD-L1 (22C3) after NAC will be necessary.

Previous studies [13–17] have reported no significant changes in TIL levels after NAC. Our study also showed no significant alteration in TIL infiltration after NAC. However, the proportion of PD-L1 (SP142)-positive immune cells increased by 1.6%, 4.1% and 2.8% after NAC in the low-, moderate-, and high-TIL groups and the change was significant in the low- and moderate-TIL groups. It seems that the degree of increase was not proportional to the quantity of TILs but relatively constant across the groups. As PD-L1 (SP142) expression is evaluated in all types of immune cells, immune cells other than lymphocytes may play a role in increase of PD-L1 (SP142) expression after NAC. Further studies are needed to investigate which type of immune cells show an increase of PD-L1 (SP142) expression after NAC.

Our study demonstrated the predictive value of positive PD-L1 status (both SP142 and 22C3) for pCR after NAC, in accordance with previous studies [13, 28, 29]. However, the prognostic value of PD-L1 status in TNBCs has not been clearly demonstrated in previous studies. Tomioka et al. [17] showed that TNBCs with combined low-TIL (< 30%) and high-tumoral-PD-L1 (SP142) (≥ 50%) status in pre-NAC were associated with unfavorable prognosis, but no case in this study met this condition. Lee et al. [15] reported that negative conversion of PD-L1 (SP263) was associated with poor disease-free survival and that positive stromal PD-L1 (SP263) expression before NAC was associated with superior disease-free survival. In this study, positive PD-L1 (SP142) status before and after NAC and an increase in PD-L1 (SP142)-positive immune cells after NAC were associated with better survival in patients with TNBC, although they were not proven to be independent prognostic factors. Taken together, PD-L1 expression in the immune cells of TNBCs seems to be associated with a favorable prognosis. The reason why positive PD-L1 status or an increase in PD-L1-positive immune cells after NAC was related to the good prognosis of patients in this study remains unclear. One possible explanation is that PD-L1 expression is associated with favorable features. In particular, an increase in PD-L1-positive immune cells after NAC correlated with a low clinical T stage, high TIL infiltration before and after NAC, negative node status after NAC, and low RCB class, all of which are favorable prognostic factors.

Although limited by the small number of patients, patients treated with platinum-based agents showed a remarkable increase, from 0.3% to 6.8%, in PD-L1 (SP142)-positive immune cells after NAC. A significant increase in PD-L1 expression after platinum-based NAC was reported in lung cancers [30]. These results indicate that PD-L1 status should be reassessed, especially after platinum-based chemotherapy, to search for possible new immunotherapy candidates. This also implies the need for new combination strategies for chemotherapy and immunotherapy. Yi et al. [31] reviewed the synergistic antitumor efficacy of combination therapies, including chemotherapy and immunotherapy. Some cytotoxic chemotherapies induce immunogenic cell death and stimulate antitumor immune responses.

The current study has the following strength: this study collected relatively large number of paired TNBC samples before and after NAC. PD-L1 and TILs were evaluated in representative whole slides, not in the tumor microarray. However, this study was limited by the inclusion of patients already evaluated for PD-L1 (SP142) for clinical purposes (thus the inclusion of non-consecutive patients with TNBC), various NAC regimens administered to the patients, and a short follow-up period with an average 2.85 years. Furthermore, response to immunotherapy was not evaluated in this study, because only small number of patients received immunotherapy. Among the patients included in this study, 5 received immunotherapy after relapse: 1 atezolizumab and 4 pembrolizumab. Six patients received neoadjuvant pembrolizumab therapy, irrespective of PD-L1 (22C3) status. Three patients with positive PD-L1 (22C3) status achieved pCR, while the others with negative PD-L1 (22C3) status revealed residual tumor after NAC. Future large studies on PD-L1 expression and immunotherapeutic outcomes are warranted.

In conclusion, PD-L1 (SP142) status changes after NAC, mostly as a positive conversion. Positive PD-L1 status before NAC is correlated with a better response to NAC. Patients with positive PD-L1 (SP142) status and increased PD-L1 (SP142)-positive immune cells after NAC show better RFS. PD-L1 (SP142) expression increases dramatically, with an average increase of > 20-fold, after treatment with the platinum agent. As PD-L1 status can convey prognostic and predictive information, it must be tested before and after NAC.

### Supplementary Information

Below is the link to the electronic supplementary material.

Supplementary file1 (DOCX 18 KB)

## Data Availability

The datasets generated during and/or analyzed during the current study are available from the corresponding author on reasonable request.
