# Supplementary material for: Alteration of PD-L1 (SP142) status after neoadjuvant chemotherapy and its clinical significance in triple-negative breast cancer
Source: Breast Cancer Res Treat. 2024 May 16;207(2):301–11. doi: 10.1007/s10549-024-07359-x (PMC11297096; doi:10.1007/s10549-024-07359-x)
Supplement: Supplementary file 1 — Supplementary file1 (DOCX 18 KB) [file 10549_2024_7359_MOESM1_ESM.docx]

**Supplementary Table S1. Clinicopathological characteristics of patients before neoadjuvant chemotherapy**

| Characteristics | No (%) |
| --- | --- |
| cT category |  |
| T1 | 15 (8.2) |
| T2 | 130 (71.4) |
| T3 | 24 (13.2) |
| T4 | 13 (7.1) |
| cN category |  |
| N0 | 65 (35.7) |
| N1 | 64 (35.2) |
| N2 | 33 (18.1) |
| N3 | 20 (11.0) |
| Clinical stage |  |
| I | 5 (2.7) |
| II | 61 (33.5) |
| III | 116 (63.7) |
| NAC regimen |  |
| Anthracycline and taxane | 144 (79.1) |
| Anthracycline, taxane and platinum agent | 14 (7.7) |
| Anthracycline | 12 (6.6) |
| Anthracycline, taxane, platinum agent and pembrolizumab | 5 (2.7) |
| Taxane and platinum agent | 6 (3.3) |
| Taxane, platinum agent and pembrolizumab | 1 (0.5) |
| Histologic subtype |  |
| Invasive carcinoma of no special type | 164 (90.1) |
| Metaplastic carcinoma | 9 (4.9) |
| Apocrine carcinoma | 3 (1.6) |
| Acinic cell carcinoma | 2 (1.1) |
| Others | 4 (2.2) |
| Histologic grade |  |
| II | 38 (20.9) |
| III | 144 (79.1) |
| Ki-67 index |  |
| <50% | 66 (36.3) |
| ≥50% | 116 (63.7) |
| TIL category |  |
| Low (TIL <10%) | 92 (50.5) |
| Moderate (10% ≤TIL <50%) | 58 (31.9) |
| High (TIL≥50%) | 32 (17.6) |

NAC, neoadjuvant chemotherapy; TIL, tumor-infiltrating lymphocytes

**Supplementary Table S2. TIL status before and after neoadjuvant chemotherapy**

| TIL category | | After NAC | | | |
| --- | --- | --- | --- | --- | --- |
|  |  | TIL<10% | 10% ≤TIL <50% | TIL≥50% | Total |
| Before NAC | TIL<10% | 55 (71.4) | 8 (28.6) | 0 (0.0) | 63 (57.8) |
|  | 10% ≤TIL <50% | 14 (18.2) | 11 (39.3) | 3 (75.0) | 28 (25.7) |
|  | TIL≥50% | 8 (10.4) | 9 (32.1) | 1 (25.0) | 18 (16,5) |
|  | Total | 77 (100.0) | 28 (100.0) | 4 (100.0) | 109 (100.0) |

Rho=0.429 (*p*<0.001)

TIL, tumor-infiltrating lymphocytes

Number in parenthesis indicate percentage.

**Supplementary Table S3. PD-L1 (SP142) and TIL category distribution before and after neo-adjuvant chemotherapy**

| PD-L1 status | | TIL<10% | 10% ≤TIL <50% | TIL ≥50% | Total |
| --- | --- | --- | --- | --- | --- |
| Before NAC (n=182) | PD-L1 negative | 71 (77.2) | 24 (41.4) | 6 (18.8) | 101 (55.5) |
|  | PD-L1 positive | 21 (22.8) | 34 (58.6) | 26 (81.3) | 81 (44.5) |
|  | Total | 92 (100.0) | 58 (100.0) | 32 (100.0) | 182 (100.0) |
| After NAC  (n=109) | PD-L1 negative | 47 (61.0) | 3 (10.7) | 0 (0.0) | 50 (45.9) |
|  | PD-L1 positive | 30 (39.0) | 25 (89.3) | 4 (100.0) | 59 (54.1) |
|  | Total | 771(100.0) | 28 (100.0) | 4 (100.0) | 109 (100.0) |

Before NAC, rho=0.467 (*p*<0.001); After NAC, rho=0.473 (*p*<0.001)

NAC, neoadjuvant chemotherapy; TIL, tumor-infiltrating lymphocytes

Number in parenthesis indicate percentage.

**Supplementary Table S4. Characteristics of tumors in relation to platinum-based chemotherapy**

| Characteristics | Platinum-based chemotherapy | | *p*-value |
| --- | --- | --- | --- |
|  | Not used (n=156) | Used  (n=26) |  |
| Clinical stage |  |  | 0.072 |
| I and II | 65 (41.7) | 6 (23.1) |  |
| III | 91 (58.3) | 20 (76.9) |  |
| Pathologic complete response |  |  | 0.898 |
| Not achieved | 104 (66.7) | 17 (65.4) |  |
| Achieved | 52 (33.3) | 9 (34.6) |  |
| Recurrence |  |  | 0.745 |
| No | 136 (87.2) | 24 (92.3) |  |
| Yes | 20 (12.8) | 2 (7.7) |  |
| PD-L1 increase |  |  | 0.268 |
| No | 60 (64.5) | 8 (50.0) |  |
| Yes | 33 (35.5) | 8 (50.0) |  |
| PD-L1 positive conversion |  |  | 0.100 |
| No | 76 (81.7) | 10 (62.5) |  |
| Yes | 17 (18.3) | 6 (37.5) |  |

Number in parenthesis indicate percentage.
*p*-value calculated by Chi-square test or Fisher’s exact test.
